# Non-suicidal self-injury in adolescents: a clinician’s guide to understanding the phenomenon, diagnostic challenges, and evidence-based treatments

**DOI:** 10.3389/fpsyt.2025.1605508

**Published:** 2025-07-29

**Authors:** Massimo Apicella, Maria Pontillo, Gino Maglio, Cristina Di Vincenzo, Giorgia Della Santa, Elisa Andracchio, Stefano Vicari

**Affiliations:** ^1^ Child and Adolescent Neuropsychiatry Unit, Bambino Gesù Children’s Hospital, Istituto di Ricovero e Cura a Carattere Scientifico (IRCCS), Rome, Italy; ^2^ Department of Neuroscience, Catholic University of the Sacred Hearth, Rome, Italy; ^3^ Department of Psychology, Catholic University of Sacred Hearth, Milan, Italy; ^4^ Department of Life Sciences and Public Health, Catholic University of the Sacred Hearth, Rome, Italy

**Keywords:** non-suicidal self-injury, suicide, adolescence, depression, mood disorders, emotion dysregulation, personality, psychotherapy

## Abstract

Non-suicidal self-injury (NSSI) is a prevalent and complex behavior, particularly among adolescents. This review explores the definition, epidemiology, and clinical significance of NSSI, emphasizing its role as a risk factor for suicidal behavior and its association with emotional dysregulation. Empirical and theoretical transdiagnostic approaches of different origins have advanced our understanding of NSSI, highlighting the importance of underlying psychological processes such as impulsivity, negative affect, and maladaptive coping strategies, yet NSSI in specific clinical presentations (e.g. Ultra-High Risk State for psychosis, autism spectrum disorders, gender dysphoria) remains understudied and less understood. Despite its inclusion in the DSM-5-TR as a condition for further study, NSSI remains undertreated, posing significant challenges for mental health care systems. The review examines the relationship between NSSI and specific psychiatric comorbidities, not limited to mood disorders, underscoring the need for tailored interventions. Psychotherapeutic approaches, such as Cognitive Behavioral Therapy (CBT), Dialectical Behavior Therapy (DBT), and Mentalization-Based Therapy (MBT), have shown promise in reducing NSSI, though long-term efficacy and comparative outcomes require further investigation. Pharmacological treatments, while limited, should focus on managing comorbid conditions rather than directly targeting NSSI. Emerging interventions may offer some potential but need further development. By integrating biological, psychological, and social perspectives, clinicians and researchers can better understand and address the multifaceted nature of NSSI. Early identification, comprehensive assessment, and targeted interventions are essential to mitigate the impact of NSSI and improve outcomes for affected individuals.

## 1 Introduction

Non-suicidal self-injury (NSSI) is defined as the deliberate, self-inflicted damage of body tissue without suicidal intent and for purposes not socially or culturally sanctioned (1). Common methods include cutting, burning, scratching, and hitting body parts, with cutting being the most frequent. NSSI poses significant clinical challenges, including identifying underlying factors, assessing psychopathology, treating comorbid disorders, and implementing safety plans. Family involvement is often crucial for comprehensive care.

## 2 Epidemiology and relevance of the problem

NSSI is highly prevalent among adolescents. A meta-analysis reports a lifetime prevalence of 23%, with higher rates in females (21.3%) than males (13.7%) (2). The peak age of onset is 12–14 years, earlier than for suicidal behavior (3). While many ceases spontaneously, approximately 20% continue for over five years (4).

NSSI is a significant risk factor for suicidal behavior, with longitudinal studies showing it predicts both concurrent and future suicide attempts (5, 6). Over half of adolescents who engage in NSSI report multiple suicide attempts (7), and it is a strong predictor of death by suicide (8). Repeated NSSI may reduce natural barriers to self-harm, increasing suicide capability, consistent with the Interpersonal Theory of Suicide (9).

NSSI remains a public health challenge, with a considerable proportion of patients hospitalized for NSSI (10). However, delays in seeking help are common, with even two-year gap between onset and receiving care (11). During the COVID-19 pandemic, self-harm presentations did not increase, suggesting reduced access to care may have led to later, more severe presentations (12–14).

## 3 Considerations on nosology

NSSI is included in the DSM-5-TR (15) as Non-suicidal Self-Injury Disorder (NSSI-D; ICD-10-CM code R45.88). Diagnostic criteria require intentional self-injury on five or more days in the past year, without suicidal intent, and significant distress or impairment. Approximately 6.7% of adolescents meet these criteria (16). However, NSSI-D as a standalone diagnosis is rare and often unstable (17), suggesting it is a precursor to broader psychopathology rather than a distinct disorder.

Included among borderline personality disorder (BPD) criteria of DSM-IV-TR (18), NSSI is frequent across various diagnoses, including mood, anxiety, trauma and stress related, substance use, and eating disorders (19). Its presence also in non-clinical populations (20) underscores its complexity. The DSM-5’s inclusion of NSSI in Section III has helped avoid the potentially inappropriate application of a personality disorder label to individuals in developmental stages, while simultaneously encouraging targeted research for this behavior, although some criticisms remain. For instance, self-poisoning is not included in the DSM-5-TR definition of NSSI, despite being a self-harming behavior that often occurs without suicidal intent (21–23).

## 4 NSSI in mood disorders versus a trans-categorical approach

NSSI is particularly prevalent among individuals with mood disorders, though its frequency varies across diagnostic categories. Research indicates that those with major depressive disorder (MDD) and bipolar disorder type II (BD-II) are more likely to engage in NSSI compared to those with bipolar disorder type I (24). Interestingly, NSSI has also been observed in prepubertal children diagnosed with bipolar disorder (25). However, the focus on specific DSM-5 (26) diagnoses may not fully capture the complexity of NSSI. Instead, transdiagnostic dimensions have emerged as stronger predictors of NSSI than categorical diagnoses. They account for a greater proportion of variance in explaining NSSI in general population samples, with DSM diagnoses offering only modest incremental predictive value (27). This underscores the importance of shifting attention from rigid diagnostic frameworks to underlying psychological and emotional processes when assessing and addressing NSSI.

## 5 Intrapersonal factors in NSSI: negative affect, cognitive processes and emotion regulation

Research on transdiagnostic factors begins with the broad concept of negative affect, which encompasses a range of distressing emotional states. NSSI has been strongly linked to feelings of loneliness (28), often exacerbated by experiences of rejection (28, 29) or anger (28). Negative thinking patterns and poor self-perception, particularly self-dissatisfaction, are frequently reported by individuals who engage in NSSI (30, 31). Traits such as perfectionism (32) and rigid thinking styles (33) have been associated with NSSI. Impulsivity, especially negative urgency—the tendency to act impulsively in response to negative emotions—has been identified as a significant and independent predictor of NSSI (34, 35). Studies examining neurocognitive performance as a risk factor for NSSI have yielded mixed and inconclusive results (36).

A significant portion of psychological research on NSSI focuses on its role in emotional regulation. Chapman et al. (37) proposed that NSSI functions as a mechanism to regulate unwanted emotional experiences, as a form of emotional avoidance. Studies using ecological momentary assessment have shown that NSSI is typically preceded by high-arousal negative emotions and followed by a reduction in these emotions and feelings of relief (28). This process, known as intrapersonal negative reinforcement, is central to the maintenance and chronicity of NSSI (38).

Nock and Prinstein’s four-function model (39, 40) provides a comprehensive framework for understanding NSSI. The model identifies four primary functions of NSSI: 1) Automatic-Negative: Using NSSI to reduce unpleasant internal states. 2) Automatic-Positive: Using NSSI to generate desirable internal states. 3) Social-Negative: Using NSSI to escape interpersonal demands. 4) Social-Positive: Using NSSI to gain attention or desirable responses from others. The Automatic-Negative function is particularly associated with greater hopelessness and an increased risk of suicidal behavior. The model also highlights that NSSI is maintained through both positive and negative self-reinforcing processes, including intrapersonal and interpersonal reinforcement.

From a cognitive neuroscience perspective, repeated engagement in NSSI can lead to habit formation, like other behaviors that provide immediate rewards. This process involves a shift in the locus of control within the striatum from the ventral to the dorsal striatum, a mechanism observed in NSSI (41), substance use disorders (42), and anorexia nervosa (43). This suggests shared neurobiological pathways across these conditions (44).

The Emotional Cascade Model (45) provides a framework for understanding NSSI through the lens of emotional regulation, also highlighting the role of rumination. The model posits that a positive feedback loop occurs between rumination and negative affect, where rumination amplifies negative emotions, and heightened negative emotions, in turn, intensify rumination. NSSI is theorized to interrupt this cascade by redirecting attention away from distressing thoughts (46). Depressive rumination itself can become habitual, often perceived as productive and thus self-reinforcing (47). Deficits in executive functioning and poor social problem-solving skills further exacerbate this cycle (48, 49).

Depressive rumination cannot be considered alone in explaining acting-out with a self-injurious behavior, and together with other depressive symptoms, high-arousal, negatively valenced emotional states —such as anxiety, tension, and anger—are particularly implicated in NSSI (50–52). Furthermore, mood reactivity and chronic irritability have been linked to self-harm (53), an observation consistently supported by clinical experience. In contrast, trait mindfulness—defined as non-judgmental attention to the present moment—has been identified as a protective factor against NSSI (54).The Benefits and Barriers Model of NSSI (55) offers additional insights. This model challenges the assumption that the relief experienced after self-inflicted pain is unique to individuals who engage in NSSI. Instead, it posits that NSSI has the potential to provide benefits for nearly everyone, but most individuals are deterred by physiological, psychological, and social barriers. Distal and proximal risk factors—such as a negative self-concept—weaken these barriers.

## 6 Focus on emotion dysregulation and NSSI

Emotional Dysregulation (ED) has emerged as a central concept in understanding NSSI within a transdiagnostic framework. It is defined as the maladaptive processing of external or internal stimuli when emotion regulation strategies are impaired. Clinically, it manifests as hyperarousal, mood instability, irritability, aggression, and temper tantrums (56). Key components of ED include heightened emotional reactivity—encompassing the threshold, intensity, and duration of emotional experiences (57)—and a failure to effectively modulate emotional states (58, 59). Gratz and Roemer (60) further demonstrated that limited access to effective emotion regulation strategies and a lack of emotional clarity predict self-injurious behaviors, particularly in females. ED has also been linked to NSSI in individuals with BD-II and MDD (24), highlighting its relevance across mood disorders. A meta-analysis (61) found a significant association between ED and NSSI, with a pooled odds ratio of 3.03. According to other authors the association is significant but smaller (OR=1.05) and ED has been suggested to be more significant as short-term rather than long-term predictor of NSSI (62).

Recent research has also explored the neurobiological underpinnings of ED in NSSI. For instance, non-adaptive cognitive emotion regulation strategies, such as shifting blame to others, have been linked to reduced activity in the Rolandic operculum and increased activity in the precentral gyrus. The Rolandic operculum, in particular, plays a role in processing external and internal sensory signals and cognitive processing of emotions (63).

A common limitation in NSSI research is the reliance on cross-sectional designs and self-report measures, which may not reliably predict subsequent NSSI (64). Despite these limitations, longitudinal studies have demonstrated that ED predicts increased NSSI behavior over both short- and long-term periods (65), underscoring its importance as a risk factor. Additionally, different ED profiles have been associated with maintaining abstinence from NSSI following non-pharmacologic treatment (66).

Linked to the ED theoretical framework, the construct of Cyclothymic Hypersensitive Temperament, characterized by intense emotional fluctuations, has been identified as a significant risk factor for NSSI (67, 68). This temperament aligns with Akiskal’s description of cyclothymia in adolescents as intermittently intense emotionality (69) and is worth further exploration in order to advance our understanding current comprehension of transdiagnostic influences and specific developmental stages versus categorial diagnosis of cyclotomic disorder.

## 7 Interpersonal factors in NSSI: adverse childhood experiences, family and peer influence

A substantial body of literature highlights the role of adverse childhood experiences (ACEs) in the development of NSSI. Emotional abuse has been strongly linked to NSSI onset, though physical neglect, physical abuse, emotional neglect, and sexual abuse have all been implicated (70).

Of note, ED is a recognized mediator between ACEs and affective symptoms (71) and between ACEs and interpersonal difficulties (72) in youth, factors which have been implied in NSSI-D development. ED also mediates between ACEs and alcohol/substance use (73, 74) and behavioral addictions (75), all factors which points toward a mediator role of ED between ACEs and NSSI, which has not yet been formally assessed.

Social factors, such as reduced contact with family and friends, as well as lower perceived social support, have also been associated with NSSI (76). Conversely, supportive parenting styles have been identified as protective factors against NSSI, emphasizing the importance of a nurturing environment in mitigating risk. Additional social factors have been implied in the reinforcement of NSSI. Perceived support after disclosing NSSI can paradoxically increase urges to self-injure, even if the individual does not explicitly recognize the connection between NSSI and social influence (77). Social learning theory (78, 79) suggests that behaviors like NSSI can be acquired through observation and imitation, particularly in peer environments characterized by impulsive and risk-taking behaviors (80).

Of great relevance, Marsha Linehan’s Biosocial Theory (81) provides a framework for understanding NSSI as a maladaptive strategy to regulate overwhelming negative emotions. The theory emphasizes the interplay between biological vulnerabilities and an invalidating social environment, which together contribute to the development of ED and, consequently, NSSI as a coping mechanism.

## 8 Focus on NSSI functions with an empirical approach

Clinical research has identified several key functions of NSSI, with affect regulation being the most commonly reported motivation (82). Other functions include anti-dissociation, anti-suicide, establishing interpersonal boundaries, interpersonal influence, self-punishment, and sensation-seeking. Factor-analytic studies have categorized these functions into two broad domains: Intrapersonal Functions (e.g., affect regulation, self-punishment) and Social Functions (e.g., interpersonal influence, establishing boundaries) (83). This categorization aligns with Nock and Prinstein’s four-function model (84).

Different NSSI functions have distinct implications for treatment and prognosis. Individuals who primarily endorse Intrapersonal Functions tend to exhibit a higher risk of suicidal behaviors and may benefit from interventions focused on improving emotion regulation skills. In contrast, those who endorse Social Functions may respond better to interventions aimed at developing effective interpersonal skills (83). A latent class analysis identified a subgroup of individuals (11% of the sample) characterized by self-cutting behaviors performed in private, primarily serving automatic functions. This subgroup exhibited the highest risk for suicidal ideation and behaviors (85).

A significant limitation of current research is the uncertainty regarding the stability of NSSI functions over time. Longitudinal studies are needed to clarify whether these classifications remain consistent. Additionally, distinguishing individuals with NSSI who are at high risk for suicidal behavior from those who are not remains a challenge (86, 87).

## 9 NSSI in specific comorbid disorders

NSSI in specific comorbidities poses specific diagnostic challenges and needs more research.

Not infrequently patients with eating disorders present to mental health services with comorbid NSSI. Shared traits between the disorders include perfectionism, alexithymia, low self-esteem, and impulsivity (88). However, a “multi-impulsive” subgroup of patients with eating disorders and high rates of comorbid NSSI has been delineated and is worth further exploration (89). Of note, ED represents a shared factor between binging/purging behaviors and NSSI (89).

Affect regulation is a shared factor between NSSI and substance use, and individuals may shift between these behaviors (90). Poor emotion regulation in adolescents is linked to marijuana use, which in turn can further disrupt emotional processing and exacerbate NSSI risk (91, 92).

Self-harm linked to delusions often differs from NSSI, as it is typically more extreme and less repetitive (93) and must be differentiated from NSSI. NSSI, however, has been suggested to be highly prevalent in Ultra-High Risk (UHR) individuals (94). Unique risk factors are reported for NSSI in this population, including higher insight and auditory hallucinations (95).

Though dissociative disorder is not a common primary diagnosis in adolescents, dissociative symptoms are prevalent among psychiatric patients with NSSI or suicidal attempts (96). NSSI in this context may serve as a coping mechanism to counteract dissociation, providing temporary relief or reconnection to reality (97).

Self-harm in Autism Spectrum Disorders (ASD) may represent either repetitive Self-Injurious Behavior (SIB) or NSSI. SIB, such as hitting or biting, is more common in severe intellectual disability (98), while DSM-5-TR NSSI-D in ASD remains understudied and may require tailored assessment tools, since measures on comorbid depressive symptoms and emotion regulation seem inadequate in this population (99).

NSSI is highly prevalent in individuals with Gender Dysphoria, often explained by the Minority Stress Model (100). Unique functions in these individuals, such as reducing gender dysphoria (101), even if are not the most frequent (102), are not captured by standard NSSI measures, highlighting the need for tailored approaches.

## 10 Treatment

Developing an effective treatment plan for children and adolescents who engage in NSSI requires a comprehensive approach that includes thorough assessment, diagnostic evaluation, and consideration of key transdiagnostic dimensions and psychosocial factors. However, the field has historically faced significant challenges, such as inconsistent terminology (e.g. “parasuicide”), small sample sizes, and heterogeneity, which complicate the interpretation of research findings (103–106).

The National Institute for Health and Care Excellence (107) guidelines recommend Dialectical Behavior Therapy for Adolescents (DBT-A) for young people with significant emotional dysregulation and frequent self-harm. Developing a crisis management plan is a pivotal component of earliest stages of treatment across the most studied therapeutic approaches of Cognitive Behavioral Therapy (CBT), Dialectical Behavior Therapy (DBT), and Mentalization-Based Therapy (MBT) (108–110).

CBT focuses on cognitive restructuring, problem-solving skills, and behavioral counseling. Evidence from trials combining CBT with antidepressants, such as fluoxetine, suggests a reduction in NSSI incidence (111). However, in a metanalysis CBT has not demonstrated superiority over other interventions for NSSI (112). NSSI is a recognized risk factor for worse outcomes of CBT also in trials for anxiety and depression (113).

DBT, originally developed by Marsha Linehan for BPD, integrates individual therapy, group skills training, and crisis management. DBT-A, a 12-week adaptation for adolescents, has shown small to moderate effects in reducing self-harm (112, 114). Long-term follow-up studies indicate that the benefits of DBT, including reductions in NSSI, are sustained over time, although general functioning may remain impaired (115).

MBT, rooted in psychodynamic attachment theory, aims to enhance the capacity to understand oneself and others in terms of intentional mental states, thereby improving self-control and emotional regulation. An adaptation for adolescents (MBT-A), which includes family sessions, has shown promising results in reducing self-harm, though evidence of its superiority over control interventions remains inconsistent (112, 116). While long-term data on all treatment for BPD and NSSI are limited, we report for one study of MBT for adults a 8-year follow-up is available, showing reduced suicide attempts with MBT (117).

Pharmacological interventions specifically targeting NSSI are scarce, and NICE guidelines (107) explicitly recommend against using medications as a primary treatment for self-harm. While some studies suggest potential benefits of medications acting on the serotonergic, dopaminergic, and opioid systems, the evidence is mixed. For example, selective serotonin reuptake inhibitors (SSRIs) have not been shown to reduce NSSI (118), while aripiprazole demonstrated some efficacy in adults with BPD in a single trial (119). Naltrexone, an opioid antagonist, has shown preliminary promise in reducing NSSI, though further research is needed to confirm these findings (120). Overall, pharmacological treatment should focus on managing comorbid psychopathology rather than directly targeting NSSI (121).Emerging treatments, such as neuromodulation techniques are also being explored, though research in the context of NSSI remains limited. Some researchers hypothesize that stimulating the right inferior frontal gyrus, which responds to anodal transcranial direct current stimulation, may have potential in reducing NSSI (44). Repetitive transcranial magnetic stimulation (rTMS), already FDA-cleared for treating depression in adults (122), has recently garnered attention for its potential role in NSSI. A study by Zhao et al. (123) demonstrated that adjunctive rTMS, when combined with SSRI treatment in adolescents with depression, improved both depressive symptoms and NSSI compared to a control group. Furthermore, in a subsequent study, Liu et al. (124) investigated rTMS as an adjunct to sertraline in adolescents experiencing a major depressive episode with NSSI. While the combined treatment appeared to further improve depressive symptoms and showed positive effects on various memory, attention, and language tests, as well as inflammatory cytokines, a specific direct effect on NSSI behavior was not described. Despite these early findings, more robust research is needed to establish the specific efficacy of neuromodulation techniques for NSSI in adolescent populations.

## 11 Conclusion: a clinician’s roadmap

Non-suicidal self-injury (NSSI) represents a complex and multifaceted phenomenon with significant implications for adolescent mental health. Its high prevalence and established role as a major risk factor for suicidal behavior necessitate a comprehensive clinical approach that extends beyond specialized psychiatric settings.

Given that many young individuals engaging in NSSI do not access specialized child and adolescent mental health services (125), there is a critical need for increased awareness and training across all levels of care. Clinicians in primary care settings play a fundamental role in early identification. They must be particularly sensitive to known risk factors for NSSI, such as negative emotionality (126) and, specifically, ED. Direct and open inquiry about NSSI, along with concurrent screening for suicide risk (127), is paramount. It is crucial to carefully distinguish suicidal from non-suicidal self-harm acts (128), while addressing both suicidal and non-suicidal self-harming ideation and behavior directly, openly, and in a non-judgmental, non-stigmatizing manner. This requires high awareness, to reduce perceived stigma for patients and mitigate clinician fear or discomfort.

A thorough and comprehensive assessment is paramount for effective management. Clinicians must recognize that NSSI frequently co-occurs with various other mental health conditions. Therefore, treatment plans must address these comorbidities comprehensively, as their effective management can significantly impact NSSI outcomes. Psychiatrists, in particular, must be highly sensitive to the presence of mood disorders, carefully distinguishing between unipolar and bipolar presentations. Individual psychological characteristics like high irritability, affective instability, and reactivity should be investigated not only as potential symptoms of a bipolar disorder but also as indicators of underlying ED or predisposing temperamental factors. It is vital to remember that NSSI does not solely equate to borderline personality disorder or to depression; other comorbidities, ranging from eating disorders to post-traumatic stress disorders, must be thoroughly evaluated and addressed. Correctly identifying diagnoses and comorbidities in the psychopathological assessment can guide clinicians to effective pharmacotherapy, given that no drug has been proven to specifically manage NSSI. Moreover, clinicians should always inquire about and address sleep issues (129) and substance use (130).

Beyond mere recognition of NSSI, clinicians and researchers must delve deeper into understanding its functions and characterizing associated intrapersonal factors such as negative affect, cognitive distortions, and impulsivity. Equally important are the relevant interpersonal dynamics, and contextual ambiental factors, including adverse childhood experiences and peer influences. Notably, these factors can also represent independent risk factors for suicidal behavior. Clinicians should be particularly attuned to indicators of thwarted belongingness, perceived burdensomeness, and hopelessness related to these states, which, alongside an acquired capability for suicide—potentially amplified by the escalation of self-injurious acts—may represent critical risk factors for future suicide attempts.

The recognition of ED as a prominent transdiagnostic factor in the majority of NSSI cases helps in understanding the utility of current therapeutic approaches that prioritize the development of emotion regulation skills. Providing patients and families with informed psycho-diagnostic feedback on these dynamics can significantly enhance treatment motivation and improve family communication. However, it is equally important not to overlook specific populations where self-damaging behaviors might be driven by distinct and unique factors, such as in cases of psychosis (full-blown or clinical high risk), dissociative disorders, ASD, or among individuals with gender dysphoria. For these underrepresented populations, innovative research designs and tailored interventions are essential to address their unique needs.

Furthermore, consistent with the most efficacious psychotherapies for NSSI, clinicians must be prepared to develop robust crisis management plans as an integral part of the initial stages of treatment for any adolescent engaging in NSSI. Evidence-based behavioral therapies, particularly DBT, involve as a core component in the initial phase establishing a therapeutic commitment agreement, referred to as a “contract.” This implies a collaboratively developed understanding between the client and therapist, outlining strategies to mitigate immediate risks associated with suicidal ideation and NSSI, enhancing the client’s agency and commitment to safety by stipulating clear protocols for managing urges.

Safety planning interventions are crucial in evidence based psychotherapies and are often integrated effectively in clinical practice. Despite some heterogeneity in components, most plans share common intervention elements (131). A crisis management plan should be co-developed by the therapist and patient, ensuring it’s personalized, realistic, and readily accessible (e.g., written or digital) during times of crisis. Crucially, the family must be informed and proactively involved. While family dynamics can sometimes represent significant risk factors for NSSI—not only through ACEs, but also through negative parent-child relationships and invalidating experiences (132)—they also constitute an invaluable resource and protective factor within the therapeutic process. Engaging families in treatment, especially in fostering understanding of ED and enhancing supportive communication, can significantly improve outcomes. Indeed, family support, cohesion, adaptability, validating experiences, warmth, and adequate parental responsiveness serve as powerful protective factors (133). This collaborative approach can transform potential family vulnerabilities into strengths, leveraging their unique position to support the adolescent in implementing the crisis plan and fostering an environment conducive to healing.

Crisis plans across DBT, CBT, and MBT have shared features (104, 110, 134). These include: early identification of warning signs; coping strategies and distraction techniques for autonomous use; activating social support; timely activation of professional/emergency contacts; environmental safety measures (limiting access to dangerous means); and reinforcing reasons for living.

Some skills like DBT’s TIPP skills (Temperature, Intense exercise, Paced breathing, Paired muscle relaxation) (135) are proper to specific models. They can often be integrated into early psychoeducational sessions. Encouraging behavioral activation is a common strategy in CBT for mood disorders which can be often integrated in this context. Enhancing problem-solving skills and cognitive restructuring from CBT, or radical acceptance (also an ACT concept, which draws from DBT), are crucial, though often implemented in more structured phases of psychotherapy. MBT, while drawing some elements from DBT in crisis planning, emphasizes a “stand-by” attitude and more reflective content to re-enter a mentalizing state, alongside a more systematic involvement of family/caregivers trained to help their child mentalize. A critical reflective moment is essential to understand why a crisis initiated. In this aspect, DBT often utilizes chain analysis, a detailed behavioral assessment tool used to identify the sequence of events, thoughts, feelings, and behaviors that lead to a target behavior (NSSI), and to point-out where interventions could have been applied. Conversely, MBT primarily relies on exploring obstacles to its central construct of mentalization to guide learning (104, 110, 134, 136).

Finally, in acute crises, admissions must be considered. In some cases also a brief admission may offer a timeout in situations of increased stress and threat, fostering self-management in a safe environment (137).

Addressing NSSI effectively requires a collective commitment to evidence-based practice, ongoing research, and compassionate care. By integrating these insights, clinicians can empower adolescents to navigate distress, fostering resilience and promoting pathways toward healthier futures.
